# Supplementary material for: A diminutive new basilosaurid whale reveals the trajectory of the cetacean life histories during the Eocene
Source: Commun Biol. 2023 Aug 10;6:707. doi: 10.1038/s42003-023-04986-w (PMC10415296; doi:10.1038/s42003-023-04986-w)
Supplement: Supplementary file 6 — Supplementary Data 3 [file 42003_2023_4986_MOESM6_ESM.zip › Supplementary Data 3/Supplementary Data 1_BTD_ASR/trait_0102_tree.plot.pdf]

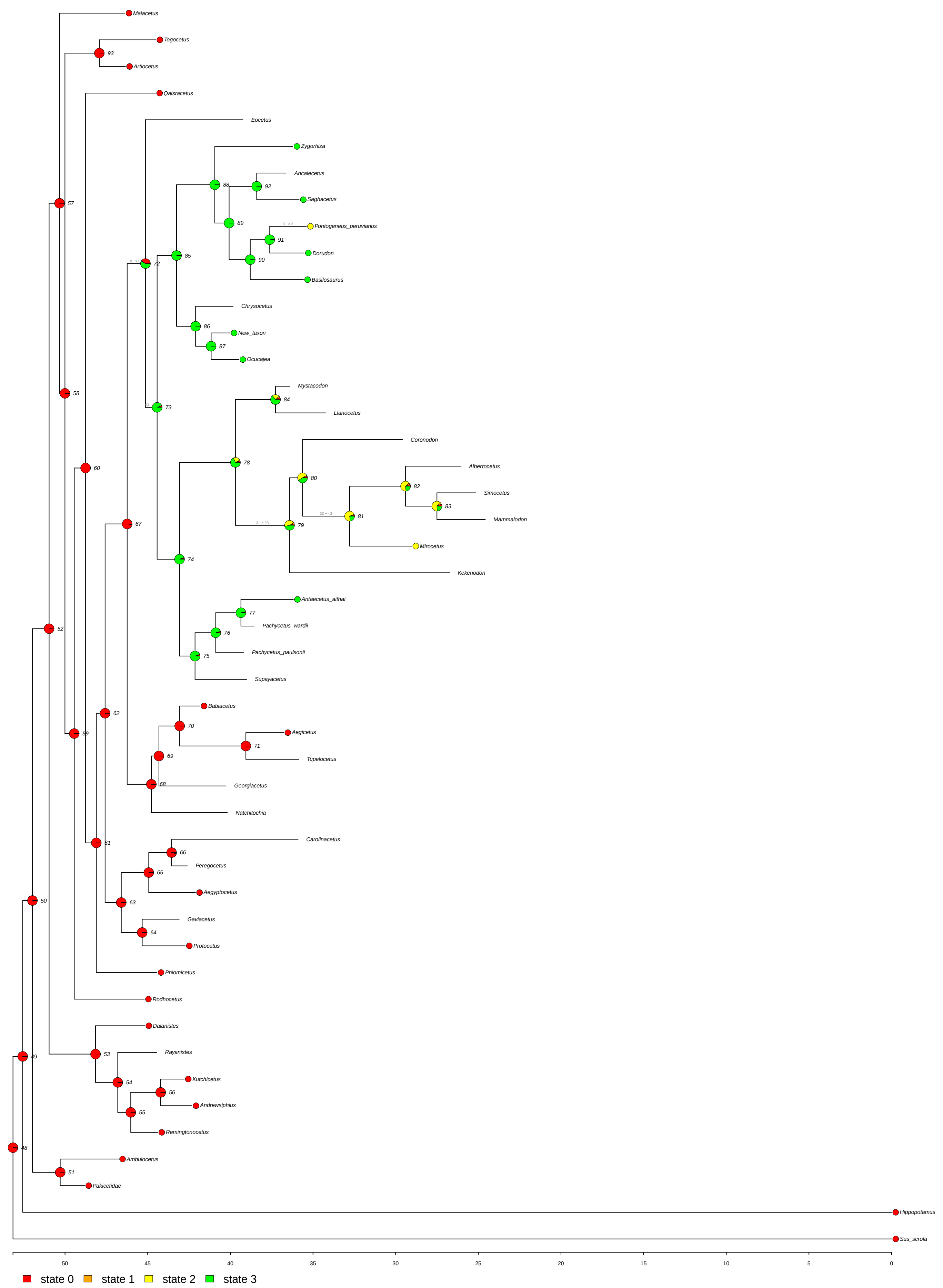
Maiacetus
Togocetus
93
Artiocetus
Qaisracetus
Eocetus
Zygorhiza
Ancalecetus
88
92
Saghacetus
89
3 -> 2
Pontogeneus_peruvianus
91
Dorudon
72
85
90
Basilosaurus
Chrysocetus
86
New_taxon
87
Ocucajea
57
Mystacodon
84
58
Llanocetus
73
Coronodon
60
78
Albertocetus
80
82
Simocetus
83
23 -> 2
81
Mammalodon
67
3 -> 23
79
Mirocetus
74
Kekenodon
Antaecetus_aithai
77
52
Pachycetus_wardii
76
Pachycetus_paulsonii
75
Supayacetus
Babiacetus
62
70
59
Aegicetus
71
Tupelocetus
69
68
Georgiacetus
Natchitochia
Carolinacetus
61
66
Peregocetus
65
50
Aegyptocetus
63
Gaviacetus
64
Protocetus
Phiomicetus
Rodhocetus
Dalanistes
49
53
Rayanistes
Kutchicetus
54
56
55
Andrewsiphius
Remingtonocetus
48
Ambulocetus
51
Pakicetidae
Hippopotamus
Sus_scrofa
50
45
40
35
30
25
20
15
10
5
0
state 0
state 1
state 2
state 3
